# Antimalarial potential of compounds isolated from *Mammea siamensis* T. Anders. flowers: in vitro and molecular docking studies

**DOI:** 10.1186/s12906-022-03742-7

**Published:** 2022-10-12

**Authors:** Prapaporn Chaniad, Arnon Chukaew, Apirak Payaka, Arisara Phuwajaroanpong, Tachpon Techarang, Walaiporn Plirat, Chuchard Punsawad

**Affiliations:** 1 School of Medicine, Walailak University, 80160 Nakhon Si Thammarat, Thailand; 2 Research Center in Tropical Pathobiology, Walailak University, 80160 Nakhon Si Thammarat, Thailand; 3 Chemistry Department, Faculty of Science and Technology, Suratthani Rajabhat University, 84100 Surat Tani, Thailand; 4 School of Science, Walailak University, 80160 Nakhon Si Thammarat, Thailand

**Keywords:** Anti-malarial activity, *Mammea siamensis*, *Plasmodium falciparum*, Molecular docking, Xanthone

## Abstract

**Background:**

The emergence of antimalarial drug resistance encourages the search for new antimalarial agents. *Mammea siamensis* belongs to the Calophyllaceae family, which is a medicinal plant that is used in traditional Thai preparations. The hexane and dichloromethane extracts of this plant were found to have potent antimalarial activity. Therefore, this study aimed to isolate active compounds from *M. siamensis* flowers and evaluate their antimalarial potential and their interactions with *Plasmodium falciparum* lactate dehydrogenase (*Pf*LDH).

**Methods:**

The compounds from *M. siamensis* flowers were isolated by chromatographic techniques and evaluated for their antimalarial activity against chloroquine (CQ)-resistant *P. falciparum* (K1) strains using a parasite lactate dehydrogenase (pLDH) assay. Interactions between the isolated compounds and the *Pf*LDH enzyme were investigated using a molecular docking method.

**Results:**

The isolation produced the following thirteen compounds: two terpenoids, lupeol (**1**) and a mixture of β-sitosterol and stigmasterol (**5**); two mammea coumarins, mammea A/AA cyclo D (**6**) and mammea A/AA cyclo F (**7**); and nine xanthones, 4,5-dihydroxy-3-methoxyxanthone (**2**), 4-hydroxyxanthone (**3**), 1,7-dihydroxyxanthone (**4**), 1,6-dihydroxyxanthone (**8**), 1-hydroxy-5,6,7-trimethoxyxanthone (**9**), 3,4,5-trihydroxyxanthone (**10**), 5-hydroxy-1-methoxyxanthone (**11**), 2-hydroxyxanthone (**12**), and 1,5-dihydroxy-6-methoxyxanthone (**13**). Compound **9** exhibited the most potent antimalarial activity with an IC_50_ value of 9.57 µM, followed by **10**, **1**, **2** and **13** with IC_50_ values of 15.48, 18.78, 20.96 and 22.27 µM, respectively. The molecular docking results indicated that **9**, which exhibited the most potent activity, also had the best binding affinity to the *Pf*LDH enzyme in terms of its low binding energy (-7.35 kcal/mol) and formed interactions with ARG109, ASN140, and ARG171.

**Conclusion:**

These findings revealed that isolated compounds from *M. siamensis* flowers exhibited antimalarial activity. The result suggests that 1-hydroxy-5,6,7-trimethoxyxanthone is a possible lead structure as a potent inhibitor of the *Pf*LDH enzyme.

## Background

Malaria remains one of the most important tropical parasitic diseases, as it results in 1–3 million deaths annually [1]. Five different species of Plasmodium parasites cause malaria in humans. Of these, *Plasmodium falciparum* is the most virulent parasite, as it results in a high mortality and morbidity rate caused approximately 229 million cases and 409,000 deaths worldwide in 2019 [2, 3]. Plasmodium infections can produce a range of clinical effects and types of malaria, including asymptomatic parasitemia, uncomplicated malaria, severe malaria, and death [4]. In the absence of an effective malaria vaccine, antimalarial drugs remain the only therapeutic method for the prophylaxis and treatment of malaria [5]. Artemisinin-based combination therapies (ACTs) have been recommended by the World Health Organization (WHO) as a first-line treatment for uncomplicated malaria in endemic countries worldwide [6]. ACTs consist of fast-acting and stable artemisinin derivatives that are coadministered with a different long-acting partner drug, which involves a different mechanism of action, to reduce the emergence of bacterial resistance and increase treatment efficacy [7]. However, *P. falciparum* has now developed mechanisms to resist most antimalarial drugs, including current ACTs [8]. The emergence and spread of ACT-resistant malaria parasites is causing an increase in malaria incidence, epidemics, and the associated morbidity and mortality [7]. As current antimalarial drugs become increasingly ineffective, there is an urgent need to develop a novel antimalarial agent to treat and control the disease. The lactate dehydrogenase enzyme from *P. falciparum* (*Pf*LDH) has been considered an important molecule and an essential drug target for malaria treatments. This enzyme catalyzes the interconversion of pyruvate to lactate in the final step of glycolysis, which is required for energy production in living cells [9]. Inhibition of this enzyme activity results in parasitic death [10].

In the continued search for new molecular targets for drug design, herbal medicine offers an interesting choice for the research and development of antimalarial drugs because plants are an excellent source of many pharmaceutical compounds [11]. *Mammea siamensis* T. Anders. is a Thai medicinal plant that is locally known as Saraphi and belongs to the family Calophyllaceae. This plant is widely distributed in Thailand, Myanmar, Laos, Cambodia, and Vietnam [12]. Its flowers have long been used in folk medicine to treat heart problems and fevers and to increase appetite [13]. This plant and its constituents have been reported to possess antiproliferative and apoptotic effects [14], aromatase inhibitory activity [15] and the ability to inhibit nitric oxide (NO) production [16]. However, there have been no reports of antimalarial activity for this plant.

In our study, the hexane and dichloromethane extracts of *M. siamensis* possessed potent antimalarial properties, with IC_50_ values of 5.32 and 5.67 µg/ml, respectively. Therefore, the compounds from this plant were isolated and investigated for antimalarial activity. Moreover, the mechanism of the isolated compound with the *Pf*LDH enzyme was also evaluated using a molecular docking technique.

## Materials and methods

### Plant materials

The *M. siamensis* flowers were collected from Surat Thani Province, Thailand, in 2020. The collection of plant materials was performed in accordance with the relevant guidelines and regulations of the Plant Varieties Protection, Department of Agriculture, Ministry of Agriculture and Cooperatives, Thailand. The plant was identified by a botanist from the School of Pharmacy at Walailak University. The voucher specimen (SMD 122006002) was deposited in the School of Medicine, Walailak University.

### General experimental procedure

The nuclear magnetic resonance (NMR) spectra were recorded in Acetone-d6 and CDCl_3_ on an Avance NEO, a Bruker Spectrometer operating at 500 MHz for ^1^ H and 125 MHz for ^13^ C. Column chromatography was carried out using silica gel (230–400 mesh, Sili Cycle Inc., Canada). All reagents were purchased from Sigma, USA. All solvents were analytical reagent grade and purchased from Labscan, Thailand.

### Extraction and isolation of compounds

The dried *M. siamensis* flowers (3.5 kg) were ground and macerated successively with three solvents of increasing polarity. The plant material was initially extracted with hexane (2 × 20 L) for one week at room temperature. The solvent extraction was filtered through Whatman No. 1 filter paper and concentrated under reduced pressure at 50 °C to obtain a yellowish viscous crude hexane extract (21.3 g). The residue was further macerated with dichloromethane and ethanol under the same conditions mentioned above to afford a brownish viscous crude containing dichloromethane (29.7 g) and ethanol (16.2 g) extracts.

A bioassay-guided isolation of antimalarial active compounds was performed. Hexane and dichloromethane, which exhibited high activities (IC_50_ = 5.32 and 5.67 µg/ml, respectively), were selected to further isolate active compounds by chromatographic techniques. The hexane extract (18.00 g) was separated by quick column chromatography (QCC) over silica gel and eluted by step-gradient elution, which starting with hexane and then increased in polarity with EtOAc to afford seven major fractions (F1-F7) based on thin-layer chromatography (TLC) profiles. Fraction 2 (3.64 g) was purified by column chromatography (CC) using 5% EtOAc in hexane as the eluent to produce compound **1** as a white crystal solid (470.10 mg) and compound **2** as a yellow oil (170.10 mg). Fraction 4 (2.13 g) was purified by CC using 10% EtOAc in hexane as an eluent to obtain six subfractions (H1-H6). Subfraction H3 (0.57 g) was purified by CC with 10% acetone in hexane to produce compound **3** as a yellow crystal (60.50 mg). Subfraction H6 (2.17 g) was purified by CC using 15% EtOAc in hexane to give five subfractions (H6A-H6E). Subfraction H6C (0.37 g) was further purified by CC using 15% acetone in hexane followed by preparative thin layer chromatography (PTLC) to produce compound **4** as a yellow oil (24.20 mg).

The dichloromethane extract (25.00 g) was subjected to QCC over silica gel by step-gradient elution starting from dichloromethane, and then the polarity was increased with EtOAc and acetone to produce 10 fractions (F1-F10) based on TLC profiles. Fraction 3 (4.17 g) was purified by QCC to produce six subfractions (C1-C6). Subtraction C3 (0.71 g) was further subjected to CC with 10% EtOAc in hexane to afford compound **5** as a white powder (45.30 mg) and compound **6** as a yellow powder (22.40 mg). Fraction 5 (2.87 g) was purified by CC to produce seven subfractions (5 A-5G). Subtraction 5B (0.48 g) was purified by CC with 15% EtOAc in hexane to produce compound **7** as a yellow powder (5.80 mg). Subtraction 5D was separated by CC with 15% EtOAc in hexane to afford compound **8** as a yellow powder (5.70 mg) and compound **9** as a yellow powder (5.30 mg).

Subfraction 5 F (0.32 g) was subjected to CC eluting with 20% EtOAc in hexane to afford three subtractions (5F1-5F3). Subtraction 5F3 (0.08 g) was further purified by CC eluting with 20% EtOAc/hexane to obtain compound **10** as a yellow powder (41.50 mg) and compound **11** as a yellow powder (22.50 mg). Fraction 8 (0.22 g) was purified by CC to give eight subfractions (8 A-8 H). Subtraction 8 C was purified by CC with 25% EtOAc in hexane to afford compound **12** as a yellow powder (57.40 mg) and compound **13** as a yellow powder (25.30 mg).

The structures of compounds **1**–**13** were elucidated by NMR analysis, and were confirmed by comparison with previously reported data in the literature.

### Parasite cultivation

The chloroquine-resistant *P. falciparum* (K1) strain was obtained from Dr. Rapatbhorn Patrapuvich, Department of Drug Research Unit for Malaria, Faculty of Tropical Medicine, Mahidol University, Thailand. *P. falciparum* was cultured according to the method of Trager and Jensen with some modifications [17]. The parasites were cultured in noninfected human red blood cells (2% hematocrit) using RPMI 1640 medium that was supplemented with 0.5% Albumax II, 10 µg/ml hypoxanthine, 2.5 µg/ml gentamicin, 4.8 mg/ml HEPES and buffered with 2 mg/ml sodium bicarbonate as described previously in our previous study [18]. All the chemicals and reagents used for culturing were purchased from Sigma–Aldrich, New Delhi, India and Gibco, Waltham, MA USA. The culture was maintained at 37 °C in a CO_2_ incubator. The culture medium was changed, and Giemsa-stained slides were prepared daily to monitor parasitemia.

### Antimalarial activity assay

The in vitro antimalarial activity of the isolated compounds was assessed on cultured *P. falciparum* using the *Plasmodium* lactate dehydrogenase (pLDH) assay described by Makler with some modifications [19]. Briefly, the compounds were individually dissolved in DMSO to obtain a stock solution of 1 mM, and the solution was then diluted to a final working concentration of 0.78–100 µM. Chloroquine and artesunate (Sigma–Aldrich, New Delhi, India) were used as positive controls. Parasitized red blood cells (2% hematocrit, 2% parasitemia) were aliquoted into a 96-well cell culture plate, and then the infected red cells were exposed. The tested compound was added to 96-well plates and incubated at 37 °C for 72 h in a CO_2_ incubator. At the end of incubation, the plates were subjected to three freeze/thaw cycles (frozen at -20 °C and thawed at 37 °C) for complete hemolysis. The lysed cells were transferred to a new 96-well plate that contained a mixture of 100 µl of Malstat reagent and 20 µl of nitroblue tetrazolium/phenazine ethosulfate solution (Calbiochem®, Sigma–Aldrich, New Delhi, India) and were incubated for 1 h in the dark. The experiments were performed in triplicate. These solutions were used to determine the activity of the lactate dehydrogenase (LDH) enzyme in the cultures. When LDH was present, a purple product was formed, and the optical density was measured using a microplate reader at a wavelength of 650 nm. The concentrations at which 50% inhibition of parasite growth (IC_50_) was calculated using a nonlinear regression curve contained in GraphPad PRISM version 6 (GraphPad Software, Inc., La Jolla, CA, USA).

### In vitro assessment of cytotoxicity

The toxicity of isolated compounds was assessed by a 3-(4,5-dimethythiazol2-yl)-2,5-diphenyl tetrazolium bromide (MTT) assay according to a previous method [20]. Briefly, Vero cells, a normal mammalian cell line or HepG2 human hepatoma cell line, were seeded into 96-well plates (10^4^ cell/ml) and incubated for 24 h at 37 °C with 5% CO_2_. Cells were then treated with various concentrations of the tested compounds ranging from 5 to 80 µM, and the final concentration of DMSO in the tested dilutions was not higher than 1% for 48 h at 37 °C with 5% CO_2_. Subsequently, MTT solution was added to each well, and the plate was incubated for 2 h in a CO_2_ incubator. The medium was then removed, and 100 µl of DMSO was added to each well. The negative control was performed using growth medium alone instead of the tested compounds, while doxorubicin (Sigma–Aldrich, New Delhi, India) was used as the positive toxic control. The assay was performed in triplicates. Finally, the optical density was determined using a microplate reader at a wavelength of 590 nm. The 50% cytotoxic concentration (CC_50_) was calculated using a nonlinear regression curve contained in GraphPad PRISM version 6 (GraphPad Software, Inc., La Jolla, CA, USA).

## Molecular docking

***Pf*LDL structure preparation**.

The crystal structure of *Pf*LDH in complex with β-nicotinamide adenine dinucleotide phosphate disodium salt (NADH) and oxamate was downloaded from the Protein Data Bank with PDB code 1LDG. The *Pf*LDH structure was prepared using AutoDock Tools. The missing residues were incorporated. All water molecules and the oxamate were removed so that a new ligand could enter the active site [21].

### Ligand molecule preparation

Fourteen compounds from *M. siamensis* were used as ligands for the docking study. The 3D structures of compounds, including artesunate and chloroquine, were generated using the HyperChem Professional 8.0 program. (Hypercube Inc., Gainesville, FL). Each structure was geometrically optimized using the semiempirical PM3 method. Subsequently, Gasteiger charges were assigned to the ligands using AutoDock Tools to model the appropriate structures for docking calculations.

### Molecular docking analysis

The binding mode and interaction of compounds from *M. siamensis* and *Pf*LDH were determined by molecular docking using the AutoDock 4.2 program (Hypercube Inc., Gainesville, FL) according to a previous method [22]. The *Pf*LDH active site was selected as the ligand binding site. A grid box was constructed of 60 × 60 × 60 Å^3^ with a grid spacing of 0.375 Å and centered on 32, 30 and 32 Å for x, y, and z, respectively. All calculations were performed using the Lamarckian genetic algorithm (LGA) method with protein-fixed and ligand-flexible molecules. The resulting docked poses with root mean-square deviations (RMSDs) less than 2.0 Å were clustered together. The lowest energy-minimized conformation of the most populated cluster was used for further analysis of the hydrogen bond interactions [22]. The 3D hydrogen bond interactions between compounds and the binding site of an enzyme were generated by the UCSF Chimera 1.14 program, and hydrophobic interactions were evaluated using the protein ligand interaction profiler (PLIP) [23].

## Results

### Identification of compounds

**Fig. 1:**
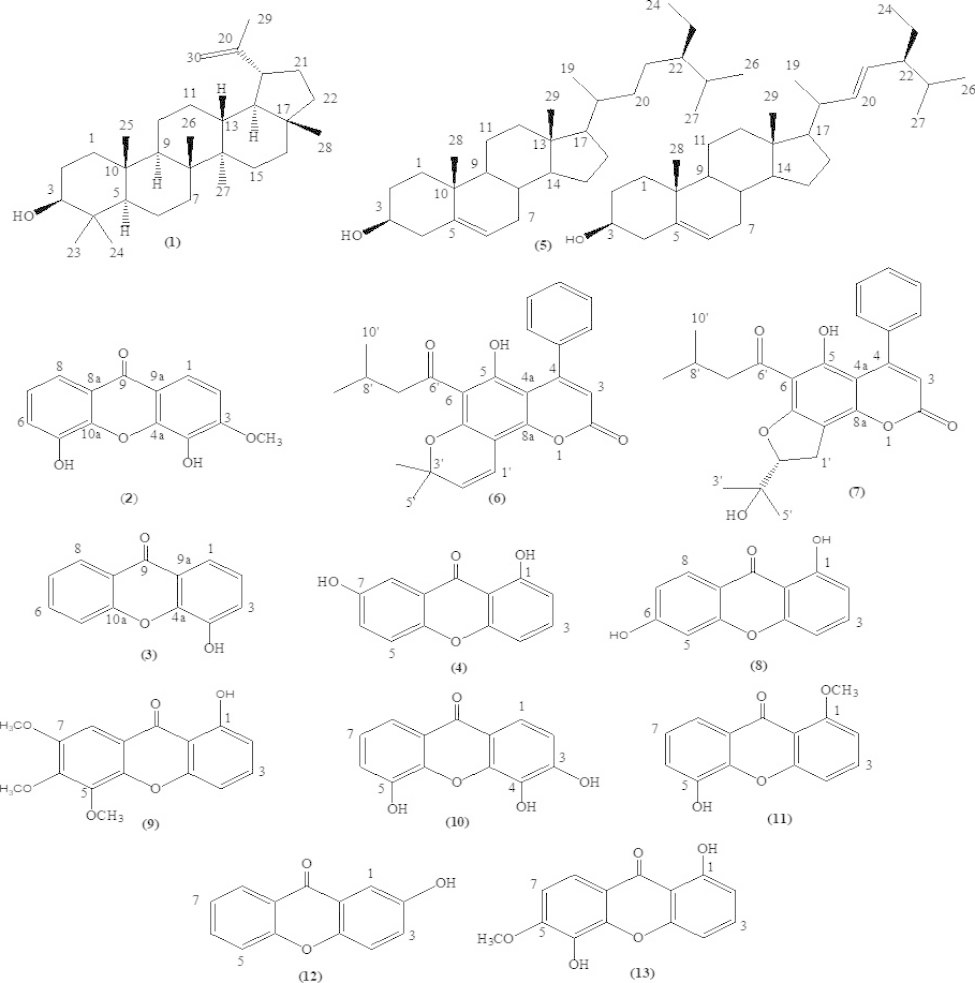
Structures of the compounds isolated from *M. siamensis* flowers

From the bioassay-guided isolation of the *M. siamensis* flower extracts, hexane and dichloromethane extracts exhibited potent antimalarial activity with IC_50_ values of 5.32 and 5.67 µg/ml, respectively. Therefore, these two extracts were selected for the isolation of active compounds. The extracts were fractionated and isolated by chromatographic techniques, and thirteen known compounds were obtained (Fig. 1). Four compounds (**1**–**4**) were isolated from the hexane extract, and nine compounds (**5**–**13**) were obtained from the dichloromethane extract. These included two terpenoids, lupeol (**1**) [24] and a mixture of β-sitosterol and stigmasterol (**5**) [25]; two mammea coumarins, mammea A/AA cyclo D (**6**) [26] and mammea A/AA cyclo F (**7**) [26]; and nine xanthones; 4,5-dihydroxy-3-methoxyxanthone (**2**) [27], 4-hydroxyxanthone (**3**) [28], 1,7-dihydroxyxanthone (**4**) [29], 1,6-dihydroxyxanthone (**8**) [30], 1-hydroxy-5,6,7-trimethoxyxanthone (**9**) [27], 3,4,5-trihydroxyxanthone (**10**) [31], 5-hydroxy-1-methoxyxanthone (**11**), 2-hydroxyxanthone (**12**) [32], and 1,5-dihydroxy-6-methoxyxanthone (**13**) [33].

### Identifications of compounds 1–13

**Lupeol (1)**: white crystal solid. ^1^ H NMR (CDCl_3_, 500 MHz): *δ* 4.59 (1 H, s, H-29a), 4.70 (1 H, s, H-29b), 3.23 (1 H, dd, *J* = 2.0, 5.8 Hz, H-3), 1.00 (3 H, s, H-23), 0.98 (3 H, s, H-24), 0.88 (3 H, s, H-25), 0.86 (3 H, s, H-26), 0.84 (3 H, s, H-27), 0.79 (3 H, s, H-28), 1.72 (3 H, s, H-30). ^13^ C NMR (CDCl_3,_ 125 MHz): *δ* 14.6 (C-27), 16.1 (C-24), 16.1 (C-26), 16.2 (C-25), 18.1 (C-28), 18.4 (C-6), 19.4 (C-30), 21.1 (C-11), 27.7 (C-2), 25.3 (C-12), 27.6 (C-15), 28.1 (C-23), 29.9 (C-21), 34.5 (C-7), 35.7 (C-16), 37.4 (C-10), 38.1 (C-1), 38.9 (C-13), 39.0 (C-4), 40.1 (C-22), 41.0 (C-8), 42.9 (C-14), 43.1 (C-17), 47.9 (C-19), 48.4 (C-18), 50.6 (C-9), 55.4 (C-5), 79.2 (C-3), 109.4 (C-29), 151.0 (C-20).

**4,5-Dihydroxy-3-methoxyxanthone (2)**: yellow oil. ^1^ H-NMR (acetone-*d*_6_, 500 MHz): *δ* 7.21 (1 H, t, *J* = 8.0 Hz, H-7), 7.30 (1 H, dd, *J* = 8.0, 1.8 Hz, H-6), 7.31 (1 H, d, *J* = 9.0 Hz, H-2), 7.41 (1 H, d, *J* = 9.0 Hz, H-1), 7.68 (1 H, dd, *J* = 8.0, 1.8 Hz, H-8), 3.93 (3 H, s, 3-OCH_3_). ^13^ C-NMR (acetone-*d*_6_, 125 MHz): *δ* 61.2 (3-OCH_3_), 113.5 (C-2), 116.1 (C-8), 116.1 (C-9a), 119.3 (C-6), 122.8 (C-8a), 123.1 (C-1), 123.3 (C-7), 144.7 (C-10a), 145.1 (C-3), 146.0 (C-5), 146.6 (C-4), 150.3 (C-4a), 175.4 (C-9).

**4-Hydroxyxanthone (3)**: yellow crystal. ^1^ H-NMR (acetone-*d*_6_, 500 MHz):* δ* 7.26 (1 H, t, *J* = 8.0 Hz, H-2), 7.36 (1 H, dd, *J* = 8.0, 1.8 Hz, H-3), 7.45 (1 H, td, *J* = 7.6, 1.5 Hz, H-7), 7.62 (1 H, dd, *J* = 7.6, 1.5 Hz, H-5), 7.71 (1 H, dd, *J* = 8.0, 1.8 Hz, H-1), 7.84 (1 H, td, *J* = 7.6, 1.5 Hz, H-6), 8.24 (1 H, dd, *J* = 7.6, 1.5 Hz, H-8). ^13^ C-NMR (acetone-*d*_6_, 125 MHz): *δ* 115.9 (C-1), 118.1 (C-5), 120.1 (C-3), 121.5 (C-8a), 122.6 (C-9a), 123.8 (C-2), 124.0 (C-7), 126.1 (C-8), 134.9 (C-6), 145.4 (C-4a), 146.6 (C-4), 155.8 (C-10a), 176.2 (C-9).

**1,7-Dihydroxyxanthone (4)**: yellow oil. ^1^ H-NMR (acetone-*d*_6_, 500 MHz): *δ* 6.77 (1 H, d, *J* = 8.3 Hz, H-2), 6.90 (1 H, d, *J* = 8.3 Hz, H-4), 7.34 (1 H, dd, *J* = 8.0, 2.6 Hz, H-6), 7.39 (1 H, d, *J* = 8.0 Hz, H-5), 7.54 (1 H, t, *J* = 8.3 Hz, H-3), 7.60 (1 H, d, *J* = 2.6 Hz, H-8), 12.66 (1 H, s, 1-OH). ^13^ C-NMR (acetone-*d*_6_, 125 MHz): *δ* 105.3 (C-8), 106.6 (C-4), 108.7 (C-9a), 110.0 (C-2), 119.0 (C-5), 120.8 (C-8a), 125.3 (C-6), 136.2 (C-3), 151.0 (C-10a), 156.0 (C-4a), 156.1 (C-7), 161.8 (C-1), 181.7 (C-9).

**Mixture of β-sitosterol and stigmasterol (2:1) (5)**: white powder.

**β-sitosterol**; ^1^ H NMR (CDCl_3_, 500 MHz): δ 5.35 (1 H, t, *J* = 6.5 Hz, H-5), 3.52 (1 H, tdd, *J* = 4.3, 4.0, 3.6 Hz, H-3), 1.00 (3 H, s, H-29), 0.92 (3 H, d, *J* = 6.3 Hz, H-19), 0.83 (3 H, t, *J* = 7.2 Hz, H-24), 0.82 (3 H, d, *J* = 6.3 Hz, H-26), 0.80 (3 H, d, *J* = 6.3 Hz, H-27), 0.67 (3 H, s, H-28). ^13^ C-NMR (CDCl_3_, 125 MHz): *δ* 12.0 (C-24), 12.4 (C-29), 19.1 (C-28), 19.4 (C-19), 19.7 (C-27), 20.3 (C-26), 21.5 (C-11), 23.5 (C-23), 26.5 (C-15), 26.5 (C-21), 28.6 (C-16), 29.6 (C-25), 32.1 (C-2), 32.2 (C-8), 32.3 (C-7), 34.3 (C-20), 36.5 (C-10), 36.8 (C-18), 37.4 (C-1), 40.1 (C-12), 42.6 (C-4), 42.8 (C-13), 46.4 (C-22), 50.4 (C-9), 56.5 (C-17), 57.1 (C-14), 72.1 (C-3), 121.9 (C-6), 141.1 (C-5).

**Stigmasterol** ; ^1^ H NMR (CDCl_3_, 500 MHz): *δ* 5.31 (1 H, t, *J* = 6.5 Hz, H-5), 3.51 (1 H, tdd, *J* = 4.3, 4.0, 3.6 Hz, H-3), 5.14 (1 H, m, H-21), 4.97(1 H, m, H-20), 1.02 (3 H, s, H-29), 0.91 (3 H, d, *J* = 6.3 Hz, H-19), 0.82 (3 H, t, *J* = 7.0 Hz, H-24), 0.81 (3 H, d, *J* = 6.5 Hz, H-26), 0.80 (3 H, d, *J* = 6.5 Hz, H-27), 0.70 (3 H, s, H-28). ^13^ C-NMR (CDCl_3_, 125 MHz): *δ* 12.2 (C-24), 12.4 (C-29), 19.0 (C-28), 19.8 (C-27), 20.4 (C-26), 21.7 (C-11), 21.9 (C-19), 24.6 (C-15), 25.5 (C-23), 29.4 (C-16), 29.7 (C-25), 31.9 (C-8), 32.1 (C-2), 32.3 (C-7), 36.7 (C-10), 37.6 (C-1), 40.0 (C-12), 40.7 (C-18), 42.3 (C-13), 42.6 (C-4), 50.2 (C-9), 56.4 (C-17), 56.9 (C-14), 72.3 (C-3), 121.7 (C-6), 129.8 (C-21), 138.9 (C-20), 141.3 (C-5).

**Mammea A/AA cyclo D (6)**: yellow powder. ^1^ H-NMR (CDCl_3_, 500 MHz): *δ* 0.96 (1 H, d, *J* = 6.6 Hz, H-9ʹ), 0.96 (1 H, d, *J* = 6.6 Hz, H-10ʹ), 1.57 (1 H, s, H-4ʹ), 1.57 (1 H, s, H-5ʹ), 2.20 (1 H, m, H-8ʹ), 2.94 (1 H, d, *J* = 6.7 Hz, H-7ʹ), 5.61 (1 H, d, *J* = 10.0 Hz, H-2ʹ), 5.96 (1 H, s, H-3), 6.87 (1 H, d, *J* = 10.0 Hz, H-1ʹ), 7.27 (1 H, m, H-3ʹʹ), 7.30 (1 H, m, H-4ʹʹ), 7.30 (1 H, m, H-5ʹʹ), 7.38 (1 H, m, H-2ʹʹ), 7.41 (1 H, m, H-6ʹʹ), 14.77 (1 H, s, 5-OH). ^13^ C-NMR (CDCl_3_, 125 MHz): *δ* 22.4 (C-9ʹ), 22.4 (C-10ʹ), 24.9 (C-8ʹ), 28.1 (C-4ʹ), 28.1 (C-5ʹ), 53.3 (C-7ʹ), 79.6 (C-3ʹ), 101.3 (C-8), 102.0 (C-4a), 107.0 (C-6), 112.5 (C-3), 115.3 (C-1ʹ), 126.1 (C-2ʹ), 126.9 (C-4ʹʹ), 127.4 (C-2ʹʹ), 127.4 (C-6ʹʹ), 128.0 (C-5ʹʹ), 128.1 (C-3ʹʹ), 139.1 (C-1ʹʹ), 154.6 (C-8a), 156.1 (C-4), 157.9(C-7), 159.4 (C-2), 164.2 (C-5), 206.5 (C-6ʹ).

**Mammea A/AA cyclo F (7)**: yellow powder. ^1^ H-NMR (CDCl_3_, 500 MHz): *δ* 0.97 (1 H, s, H-9ʹ), 0.97 (1 H, s, H-10ʹ), 1.31 (1 H, s, H-5ʹ), 1.43 (1 H, s, H-4ʹ), 2.23 (1 H, m, H-8ʹ), 2.97 (2 H, dd, *J* = 15.3, 9.0 Hz, H-7ʹ), 3.32 (1 H, d, *J* = 9.0 Hz, H-1ʹ), 4.90 (1 H, t, *J* = 9.0 Hz, H-2ʹ), 5.94 (1 H, s, H-3), 7.32 (1 H, m, H-3ʹʹ), 7.32 (1 H, m, H-4ʹʹ), 7.32 (1 H, m, H-5ʹʹ), 7.42 (1 H, m, H-2ʹʹ), 7.42 (1 H, m, H-6ʹʹ). ^13^ C-NMR (CDCl_3_, 125 MHz): *δ* 22.6 (C-9ʹ), 22.6 (C-10ʹ), 24.8 (C-5ʹ), 25.0 (C-8ʹ), 26.1 (C-4ʹ), 26.7 (C-1ʹ), 51.8 (C-7ʹ), 71.5 (C-3ʹ), 92.7 (C-2ʹ), 102.4 (C-4a), 103.2 (C-6), 104.9 (C-8), 112.0 (C-3), 127.0 (C-6ʹʹ), 127.1 (C-2ʹʹ), 127.4 (C-5ʹʹ), 127.5 (C-3ʹʹ), 128.1 (C-4ʹʹ), 138.9 (C-1ʹʹ), 155.5 (C-8a), 156.4 (C-4), 159.5 (C-2), 164.0 (C-5), 164.3 (C-7), 204.9 (C-6ʹ).

**1,6-Dihydroxyxanthone (8)**: yellow powder. ^1^ H-NMR (acetone-*d*_6_, 500 MHz):* δ* 6.77 (1 H, d, *J* = 8.0 Hz, H-2), 7.00 (1 H, d, *J* = 8.0 Hz, H-4), 7.43 (1 H, dd, *J* = 9.0, 3.0 Hz, H-7), 7.52 (1 H, d, *J* = 9.0 Hz, H-8), 7.61 (1 H, d, *J* = 3.0 Hz, H-5), 7.70 (1 H, t, *J* = 8.0 Hz, H-3). ^13^ C-NMR (acetone-*d*_6_, 125 MHz): *δ* 106.7 (C-4), 108.3 (C-5), 109.2 (C-9a), 109.3 (C-2), 119.2 (C-8), 120.8 (C-8a), 124.3 (C-7), 136.8 (C-3), 150.1 (C-6), 154.1 (C-10a), 156.4 (C-4a), 162.3 (C-1), 181.9 (C-9).

**1-Hydroxy-5,6,7-trimethoxyxanthone (9)**: yellow powder. ^1^ H-NMR (acetone-*d*_6_, 500 MHz): *δ* 6.95 (1 H, d, *J* = 8.2 Hz, H-2), 7.13 (1 H, d, *J* = 8.2 Hz, H-4), 7.20 (1 H, s, H-8), 7.68 (1 H, t, *J* = 8.2 Hz, H-3), 12.70 (1 H, brs, 1-OH), 3.96 (3 H, s, 5-OCH_3_), 3.93 (3 H, s, 6-OCH_3_), 3.95 (3 H, s, 7-OCH_3_). ^13^ C-NMR (acetone-*d*_6_, 125 MHz): *δ* 54.3 (5-OCH_3_), 55.4 (7-OCH_3_), 60.1 (6-OCH_3_), 96.3 (C-8), 105.5 (C-4), 109.5 (C-2), 111.6 (C-9a), 118.4 (C-8a), 134.3 (C-3), 139.0 (C-5), 140.1 (C-10a), 141.3 (C-6), 150.0 (C-7), 157.8 (C-4a), 160.6 (C-1), 174.1 (C-9).

**3,4,5-Trihydroxyxanthone (10)**: yellow powder. ^1^ H-NMR (acetone-*d*_6_, 500 MHz): *δ* 7.22 (1 H, t, *J* = 8.1 Hz, H-7), 7.29 (1 H, dd, *J* = 8.1, 1.8 Hz, H-6), 7.30 (1 H, d, *J* = 9.0 Hz, H-2), 7.42 (1 H, d, *J* = 9.0 Hz, H-1), 7.69 (1 H, dd, *J* = 8.1, 1.8 Hz, H-8). ^13^ C-NMR (acetone-*d*_6_, 125 MHz): *δ* 113.7 (C-2), 116.0 (C-8), 116.2 (C-9a), 119.3 (C-6), 122.9 (C-8a), 123.0 (C-1), 123.3 (C-7), 144.7 (C-10a), 145.2 (C-3), 146.0 (C-5), 146.6 (C-4), 150.2 (C-4a), 175.4 (C-9).

**5-Hydroxy-1-methoxyxanthone (11)**: yellow powder. ^1^ H-NMR (acetone-*d*_6_, 500 MHz): *δ* 6.94 (1 H, d, *J* = 8.3 Hz, H-2), 7.12 (1 H, d, *J* = 8.3 Hz, H-4), 7.19 (1 H, t, *J* = 7.9 Hz, H-7), 7.26 (1 H, dd, *J* = 7.9, 1.6 Hz, H-6), 7.62 (1 H, dd, *J* = 7.9, 1.6 Hz, H-8), 7.70 (1 H, t, *J* = 8.3 Hz, H-3), 3.94 (3 H, s, 1-OCH_3_), 9.24 (1 H, brs, 5-OH). ^13^ C-NMR (acetone-*d*_6_, 125 MHz): *δ* 55.5 (1-OCH_3_), 105.8 (C-4), 109.5 (C-2), 112.0 (C-9a), 115.8 (C-8), 119.1 (C-6), 123.3 (C-7), 123.9 (C-8a), 135.1 (C-3), 144.1 (C-10a), 145.7 (C-5), 157.5 (C-4a), 160.6 (C-1), 174.7 (C-9).

**2-Hydroxyxanthone (12)**: yellow powder. ^1^ H-NMR (acetone-*d*_6_, 500 MHz):* δ* 7.36 (1 H, dd, *J* = 8.7, 2.8 Hz, H-3), 7.43 (1 H, td, *J* = 7.6, 1.3 Hz, H-7), 7.51 (1 H, d, *J* = 8.7 Hz, H-4), 7.54 (1 H, dd, *J* = 7.6, 1.3 Hz, H-5), 7.61 (1 H, d, *J* = 2.8 Hz, H-1), 7.81 (1 H, td, *J* = 7.6, 1.3 Hz, H-6), 8.23 (1 H, dd, *J* = 7.6, 1.3 Hz, H-8), 8.90 (1 H, s, 2-OH). ^13^ C-NMR (acetone-*d*_6_, 125 MHz): *δ* 114.2 (C-1), 123.2 (C-5), 124.5 (C-4), 126.2 (C-8a), 127.5 (C-9a), 128.8 (C-7), 129.3 (C-3), 131.3 (C-8), 139.9 (C-6), 155.2 (C-4a), 159.1 (C-2), 161.3 (C-10a), 181.1 (C-9).

**1,5-Dihydroxy-6-methoxyxanthone (13)**: yellow powder, ^1^ H-NMR (acetone-*d*_6_, 500 MHz): *δ* 6.53 (1 H, d, *J* = 8.3 Hz, H-2), 6.79 (1 H, d, *J* = 8.8 Hz, H-7), 6.81 (1 H, d, *J* = 8.3 Hz, H-4), 7.45 (1 H, t, *J* = 8.3 Hz, H-3), 7.61 (1 H, d, *J* = 8.8 Hz, H-8), 12.62 (1 H, s, 1-OH), 3.79 (3 H, s, 6-OCH_3_). ^13^ C-NMR (acetone-*d*_6_, 125 MHz): *δ* 60.8 (6-OCH_3_), 106.6 (C-4), 107.8 (C-9a), 110.1 (C-2), 113.7 (C-7), 114.2 (C-8a), 121.1 (C-8), 134.5 (C-5), 136.3 (C-3), 150.9 (C-10a), 155.9 (C-4a), 156.7 (C-6), 161.8 (C-1), 181.1(C-9).

### Antimalarial activity against *P. falciparum*

The antimalarial activity of isolated compounds **1**–**13** was determined against the *P. falciparum* K1 strain by the pLDH assay and was compared with chloroquine and artesunate, a positive control (Table 1). Among these compounds, 1-hydroxy-5,6,7-trimethoxyxanthone (**9**) exhibited the highest effect with good activity (IC_50_ = 9.57 µM), followed by 3,4,5-trihydroxyxanthone (**10**, IC_50_ = 15.48 µM) and lupeol (**1**, IC_50_ = 18.78 µM), while 4,5-dihydroxy-3-methoxyxanthone (**2**), 1,5-dihydroxy-6-methoxyxanthone (**13**), and 5-hydroxy-1-methoxyxanthone (**11**) also exhibited antimalarial effects with moderate activity with IC_50_ values of 20.96, 22.27 and 29.32 µM, respectively (Table 1); however, the other compounds appeared to have weak activity, with IC_50_ values ranging from 41.67 to 74.97 µM.

**Table 1:** Antimalarial activity against *P. falciparum* and the cytotoxicity of compounds from *M. siamensis*

Compound	IC_50_ (µM)	CC_50_ (µM)	
	**K1**	**Vero cell**	**HepG2**
Lupeol (**1**)	18.78 ± 3.45^a,b^	> 80	29.27 ± 1.47^a,b^
4,5-Dihydroxy-3-methoxylxanthone (**2**)	20.96 ± 3.56^a,b^	> 80	> 80
4-Hydroxyxanthone (**3**)	68.55 ± 2.54^a,b^	48.13 ± 1.68 ^a,b^	46.12 ± 1.66 ^a,b^
1,7-Dihydroxyxanthone (**4**)	41.67 ± 2.23^a,b^	7.90 ± 0.90	13.27 ± 1.12 ^a,b^
Mixture of β-sitosterol and stigmasterol (**5**)	45.00 ± 3.51^a,b^	> 80	> 80
Mammea A/AA cyclo D (**6**)	45.62 ± 3.12^a,b^	> 80	> 80
Mammea A/AA cyclo F (**7**)	49.89 ± 0.42^a,b^	> 80	> 80
1,6-Dihydroxyxanthone (**8**)	47.94 ± 5.16^a,b^	> 80	> 80
1-Hydroxy-5,6,7-trimethoxyxanthone (**9**)	9.57 ± 1.59 ^a,b^	> 80	> 80
3,4,5-Trihydroxyxanthone (**10**)	15.48 ± 2.63^a,b^	> 80	> 80
5-Hydroxy-1-methoxyxanthone (**11**)	29.32 ± 4.44^a,b^	> 80	> 80
2-Hydroxyxanthone (**12**)	74.97 ± 0.88 ^a,b^	> 80	> 80
1,5-Dihydroxy-6-methoxyxanthone (**13**)	22.27 ± 1.67^a,b^	> 80	30.54 ± 1.49 ^a,b^
Chloroquine*	103.2 ± 4.50	ND	ND
Artesunate*	0.53 ± 0.04	ND	ND
Doxorubicin	ND	1.46 ± 0.16	1.11 ± 0.05

ND = not determined

^a^Statistically significant difference between chloroquine and the compound, *p* < 0.05 (mean ± S.D. of three determinations)

^b^Statistically significant difference between artesunate and the compound, *p* < 0.05 (mean ± S.D. of three determinations)

*Concentration of positive control with IC_50_ unit expressed in nM

### In vitro **cytotoxicity**

Ideally, in order for a drug to be considered good, it should not exhibit any undesirable side effects on normal cells [34]. The cytotoxicity of isolated compounds on Vero cell lines and HepG2 cells was evaluated by the MTT assay. The results indicated that most compounds exhibited nontoxic effects against Vero cells at a concentration of 80 µM except for compounds **3** and **4**, which achieved CC_50_ values of 48.13 and 7.90 µM, respectively. Compounds **1**, **3**, **4** and **13** exhibited a cytotoxic effect against human HepG2 hepatoma cells with CC_50_ values of 29.27, 46.12, 13.27 and 30.54 µM, respectively.

### Molecular docking

**Fig. 2:**
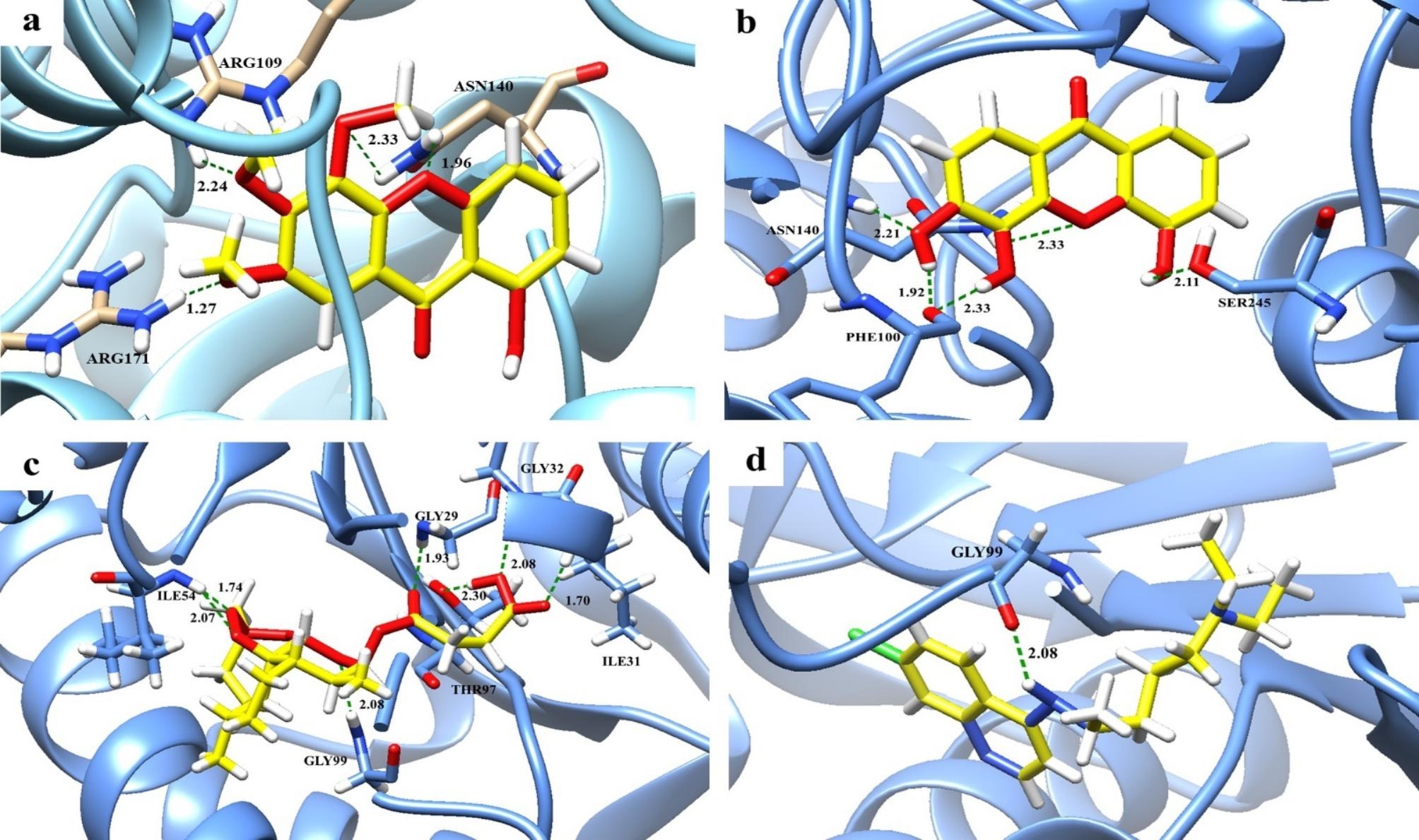
Predicted binding modes and H-bond interactions of two active compounds, artesunate and chloroquine, with the *Pf*LDH enzyme. The backbone of the *Pf*LDH enzyme is presented in a blue ribbon model, and all hydrogen bonding residues are shown as stick models and labeled by heteroatoms. The compounds are labeled by heteroatoms as follows: yellow for C, white for H, cyan for N, red for O, and green for Cl. Green dashed lines represent hydrogen bond interactions and represent bond length in angstroms (Å). a: 1-Hydroxy-5,6,7-trimethoxyxanthone (**9**), b: 3,4,5-Trihydroxyxanthone (**10**), c: Artesunate, d: Chloroquine

The binding energy and amino acid residues of *Pf*LDH that interacted with each compound, hydrogen bonds, and hydrophobic interactions are given in Table 2. To predict the binding modes of active compounds with *Pf*LDH and identify the interacting amino acid residues, the 2D interactions of two of the most active compounds (**9** and **10)** with *Pf*LDH were created, as shown in Fig. 2. Among thirteen compounds, 1-hydroxy-5,6,7-trimethoxyxanthone (**9**), which possessed the most potent antimalarial property against *P. falciparum* (IC_50_ = 9.57 µM), exhibited the best binding affinity to *Pf*LDH in terms of its low binding energy of -7.35 kcal/mol, which was the highest observed affinity to an enzyme; however, its affinity was lower than that of artesunate, which had a binding energy of -8.57 kcal/mol. Four hydrogen bonds between the amino group of ARG109, ASN140, and ARG171 interacted with compound **9.** An oxygen atom of the methoxy group at C5, C6, and C7 were responsible for these polar interactions (Fig. 2a). Additionally, this compound was stabilized through hydrophobic interactions with residues VAL26, PHE52, ILE54, ALA98, ILE119 and ILE123 (Table 2). 3,4,5-Trihydroxyxanthone (**10**), which also possessed good activity (IC_50_ = 15.48 µM), showed a remarkable binding affinity to *Pf*LDH with a binding energy of -7.25 kcal/mol. It strongly interacted with PHE100, ASN140 and SER245. All three hydroxyl groups in this compound interacted with those residues, and double hydrogen bonds were observed for PHE100 and ASN140 (Fig. 2b). Additionally, the compound forms hydrophobic interactions with residues VAL138, LEU167, PRO246 and PRO250. However, these two active compounds (**9** and **10**) interacted with amino acids lower than artesunate, which formed six hydrogen bonds, and interacted with GLY29, ILE31, GLY32, ILE54, THR97, and GLY99 of the *Pf*LDH active site **(**Fig. 2c). Chloroquine formed only one hydrogen bond with GLY99 and had a binding energy of -6.26 kcal/mol. (Fig. 2d)

**Table 2:** The binding energy and interacting amino acid residues of the *Pf*LDH with compounds from *M. siamensis*

Compound	Binding energy (kcal/mol)	H-bond interaction		Hydrophobic interaction	
		**Number of** **interaction**	**Amino acid residues**	**Number of** **interaction**	**Amino acid residues**
Lupeol (**1**)	-7.21		-	6	ILE54**, VAL55**, PHE100, ILE119
4,5-Dihydroxy-3-methoxylxanthone (**2**)	-7.14	4	PHE100, ASN140*, SER245	6	VAL138, LEU167, ALA236, PRO246*, PRO250
4-Hydroxyxanthone (**3**)	-6.31	1	TYR85	6	VAL26, PHE52, ILE54, ALA98, ILE119*
1,7-Dihydroxyxanthone (**4**)	-7.17	3	PHE100, ASN140, SER245	4	ILE31, VAL138, THR139, PRO250
β-sitosterol (**5**)	-6.45	1	PRO246	6	THR101*, LEU167, PRO246, PRO250*
Stigmasterol (**5**)	-6.64	2	ARG109, ARG171	10	MET30, ILE31*, ILE54*, ALA98, THR101, ILE119**
Mammea A/AA cyclo D (**6**)	-7.02	1	GLY29	7	PHE52, ILE54*, VAL55, ALA98, ILE119*
Mammea A/AA cyclo F (**7**)	-7.16	2	THR97, ASN140	8	MET30, ILE31*, THR101, THR139, ASN140, LEU167, PRO250
1,6-Dihydroxyxanthone (**8**)	-7.09	4	ASP53, ILE54, TYR85, GLU122	8	VAL26, PHE52*, ASP53, ILE54, ALA98, ILE119*
1-Hydroxy-5,6,7-trimethoxyxanthone (**9**)	-7.35	4	ARG109, ASN140*, ARG171	6	VAL26, PHE52, ILE54, ALA98, ILE119, ILE123
3,4,5-Trihydroxyxanthone (**10**)	-7.25	5	PHE100*, ASN140*, SER245	4	VAL138, LEU167, PRO246, PRO250
5-Hydroxy-1-methoxyxanthone (**11**)	-7.01	2	ILE54, TYR85	8	VAL26, PHE52, ILE54*, ALA98, ILE119*, ILE123
2-Hydroxyxanthone (**12**)	-6.14	2	PHE100, ASN140	4	ILE31, VAL138, THR139, PRO250
1,5-Dihydroxy-6-methoxyxanthone (**13**)	-7.14	3	ILE54, TYR85, ILE199	10	VAL26, PHE52*, ASP53, ILE54*, ALA98, ILE119*, ILE123
Artesunate	-8.57	7	GLY29, ILE31, GLY32, ILE54*, THR97, GLY99	9	VAL26, ILE31, ILE54, ALA98*, THR101, ILE119*
Chloroquine	-6.26	1	GLY99	6	VAL26, ILE31*, PHE52, THR101, ILE119

*Two interactions with amino acid residues

**Three interactions with amino acid residues

Lupeol (**1**), a terpenoid compound, showed a remarkable binding affinity to *Pf*LDH with a binding energy of -7.21 kcal/mol. It does not interact with any enzyme residue but forms hydrophobic interactions with residues ILE54, VAL55, PHE100, and ILE119. 4,5-Dihydroxy-3-methoxyxanthone (**2**), a moderately active compound, interacted with PHE100, ASN140 and SER245. A double hydrogen bond between ASN140 and the oxygen at O10 and the methoxy group at C3 was observed for this compound. This compound also forms hydrophobic interactions with VAL138, LEU167, ALA236, PRO246 and PRO250. 1,5-Dihydroxy-6-methoxyxanthone (**13**) had a lower binding energy (-7.14 kcal/mol) and formed three hydrogen bonds with ILE54, TYR85 and ILE199. It formed additional hydrophobic interactions with VAL26, PHE52, ASP53, ILE54, ALA98, ILE119, and ILE123. 5-Hydroxy-1-methoxyxanthone (**11**) showed a binding affinity to *Pf*LDH with a binding energy of -7.01 kcal/mol and formed two hydrogen bonds with ILE54 and TYR85. It also forms hydrophobic interactions with residues VAL26, PHE52, ILE54, ALA98, ILE119 and ILE123.

## Discussion

Isolating *M. siamensis* flowers led to thirteen compounds, including two terpenoids, two mammea coumarins, and nine xanthones. Xanthones (dibenzo-gamma-pirone), which were the most commonly found compounds in this study, are naturally secondary metabolite compounds that can be found in diverse terrestrial and marine plants, fungi, and lichen [35]. Xanthones belong to the class of oxygenated heterocycles, and their biological and pharmacological activities are well known in phytomedicine and medicinal chemistry [36]. Xanthone derivatives have been reported to possess a wide range of biological properties, including antioxidant, anti-inflammatory, anticancer, antidiabetic, antihypertensive, anticonvulsant, and antimalarial activities [37]. The biological activities of xanthones are associated with their tricyclic scaffold but vary depending on the nature and/or position of the different substituents [38].

Among nine xanthone compounds in the present study (**2**, **3**, **4**, **8, 9**, **10**, **11**, **12**, **13**), the results showed that they possessed a wide range of antimalarial effects that ranged from 9.57 to 74.97 µM. 1-Hydroxy-5,6,7 trimethoxyxanthone (**9**), which contains one hydroxyl group and three methoxy groups, showed the highest activity (IC_50_ = 9.57 µM). 3,4,5-Trihydroxyxanthone (**10)**, containing three hydroxyl groups, also possessed good activity (IC_50_ = 15.48 µM), whereas 4-hydroxyxanthone (**3**) and 2-hydroxyxanthone (**12**), which contain only one hydroxyl group, possessed weak activity (IC_50_ = 68.55 and 74.97 µM, respectively). Remarkably, 1,7-dihydroxyxanthone (**4**) and 1,6-dihydroxyxanthone (**8**), which contain two hydroxyl groups, appeared to have comparable activities. The results clearly revealed that the increase in the hydroxyl group becomes more frequent with **10** compared with **3** and **12**. Interestingly, the presence of a methoxy group also increased the antimalarial effect, as observed from the comparison of 5-hydroxy-1-methoxyxanthone (**11**, IC_50_ = 29.32 µM) and 4-hydroxyxanthone (**3**, IC_50_ = 68.55 µM) and as clearly observed in 1-hydroxy-5,6,7-trimethoxyxanthone **(9**, IC_50_ = 9.57 µM), the strongest active compound bearing three methoxy groups, when compared with **11**, which bears only one hydroxyl and methoxy group.

In particular, compounds **12**, **4**, **10** and **9**, which contained one, two, three hydroxyl groups, and three methoxy groups, respectively, exhibited varying antimalarial activities. Compounds that contained a high number of hydroxyl and methoxy groups exhibited more potent activity. The results clearly show the structure-activity relationship in which the number of hydroxyl and methoxy groups is associated with their biological activity. Polyhydroxylated and polymethoxylated xanthones were crucial for producing an effective compound against the *P. falciparum* K1 strain.

Regarding the antimalarial activity, our study is in accordance with previous studies in which xanthone and hydroxyxanthone derivatives presented antimalarial effect. 2,3,4,5,6-Pentahydroxyxanthone inhibits the in vitro growth of both CQ-sensitive and multidrug-resistant strains of *P. falciparum.* It exerted their antimalarial action by preventing hemozoin formation [39]. For hydroxyxanthone derivatives, 1,6,8-trihydroxyxantone exhibited the antiplasmodial activity and also inhibited heme polymerization [40].

Docking small molecule compounds into the binding site of a receptor and estimating the binding affinity of the complex are important parts of the structure-based drug design process [41]. Therefore, the present study also docked *M. siamensis* compounds with the *Pf*LDH enzyme to predict their binding mode and identify potential interactions using the molecular docking technique. The *Pf*LDH enzyme plays an essential role in controlling energy production in Plasmodium. It catalyzes the final step in the glycolytic pathway during its anaerobic erythrocytic stages of development within the human host [42]. For molecular docking, this technique is one of the most frequently used methods to predict ligand–protein interactions in structure-based drug design because of its ability to predict with a substantial degree of accuracy [43].

The docking results in accordance with the in vitro study showed that compounds **9** and **10**, which exhibited good antimalarial effects against *P. falciparum*, also strongly interacted with *Pf*LDH with a high binding energy (-7.35 and − 7.25 kcal/mol, respectively) and formed many interactions. Methoxy and hydroxyl groups of these compounds play an important role in forming hydrogen bonds. For compound **9**, all three methoxy groups interacted with ARG109, ASN140, and ARG171. For compound **10**, all three hydroxyl groups extensively formed five hydrogen bonds with PHE100, ASN140, and SER245.

In compounds that consist of only one hydroxyl group, which result in a weak antimalarial effect based on the in vitro results, the predicted hydrogen bond interactions with *Pf*LDH were weaker interactions than that of polyhydroxylated compounds in terms of binding energy. An interesting result was observed with compound **9**, which contains one hydroxyl group but has three methoxy groups, and it showed relatively strong affinity with the enzyme in terms of lower binding energy.

In the case of mammea coumarins, mammea A/AA cyclo D (**6**) and mammea A/AA cyclo F (**7**), the docking results were also in accordance with the in vitro results. These compounds interacted with *Pf*LDH with a low binding energy and formed only one and two hydrogen bonds for **6** and **7**, respectively.

The docking results supported the in vitro antimalarial activity in which the hydroxyl and methoxy groups on rings A and B of xanthone are considered to exert their antimalarial activity. They are a potential functional group for binding *Pf*LDH active sites, resulting in inhibitory action against *P. falciparum*. The results clearly showed that the presence of polyhydroxyl and polymethoxy groups enhances the antimalarial activity of the compound, as evidenced by its in vitro activity and binding energy. The active xanthone compounds interacted with PHE100, ARG109, ASN140, ARG171, and SER245 of the *Pf*LDH active site, suggesting that these amino acids are key residues for the active inhibitors, and they possibly result in interferences with the energy production process of *P. falciparum.*

Regarding other *P. falciparum* molecular targets, *P. falciparum* dihydrofolate reductase-thymidylate synthase (DHFR-TS) and *Pf*ATP6, the SERCA-type Ca^2+^-ATPase enzyme present in the malarial parasite have been proven to be major molecular drug targets of antimalarial drugs [18]. For DHFR-TS, it is a key enzyme in the folate biosynthetic pathway that is targeted by antifolates [44]. The active xanthone compounds showed the formation of a binding interaction between the compounds with the amino acids of DHFR-TS including ALA16, SER108, PHE58, ASP54 and LEU46, which is the crucial amino acids for antimalarial activity [45].

## Conclusion

These findings revealed that isolated compounds from *M. siamensis* flowers exhibited antimalarial activity. Xanthones are potent compounds that are responsible for the antimalarial activity of this plant. The findings suggest that 1-hydroxy-5,6,7-trimethoxyxanthone could be a promising lead structure as a potent inhibitor of the *Pf*LDH enzyme. Further in vivo studies are necessary to determine the efficacy of the compounds and their toxicities.

## Data Availability

The data associated to this study are included in this published article. The additional files are available from the corresponding author upon reasonable request.
